# Distinct Functions of Acyl/Alkyl Dihydroxyacetonephosphate Reductase in Peroxisomes and Endoplasmic Reticulum

**DOI:** 10.3389/fcell.2020.00855

**Published:** 2020-09-11

**Authors:** Masanori Honsho, Megumi Tanaka, Raphael A. Zoeller, Yukio Fujiki

**Affiliations:** ^1^Medical Institute of Bioregulation, Kyushu University, Fukuoka, Japan; ^2^Graduate School of Systems Life Sciences, Kyushu University, Fukuoka, Japan; ^3^Department of Physiology and Biophysics, Boston University School of Medicine, Boston, MA, United States

**Keywords:** plasmalogen, acyl/alkyl dihydroxyacetonephosphate reductase, peroxisome, endoplasmic reticulum, organelle targeting

## Abstract

Plasmalogens are a subclass of ether glycerophospholipids characterized by a vinyl-ether bond at the *sn*-1 position of the glycerol backbone. Plasmalogen biosynthesis is initiated in peroxisomes. At the third step of plasmalogen synthesis, alkyl-dihydroxyacetonephosphate (DHAP) is enzymatically reduced to 1-alkyl-*sn*-glycero-3-phospate by acyl/alkyl DHAP reductase (ADHAPR), whose activity is found in both peroxisomes and microsomes. We herein show that knockdown of *ADHAPR* in HeLa cells reduced the synthesis of ethanolamine plasmalogen (PlsEtn), similar to the Chinese hamster ovary cell mutant FAA.K1B deficient in ADHAPR activity. Endogenous ADHAPR and ectopically expressed FLAG-tagged ADHAPR were localized to peroxisomes and endoplasmic reticulum (ER) as a type I integral membrane protein in HeLa cells. ADHAPR targets to peroxisomes via a Pex19p-dependent class I pathway. In addition, it is also inserted into the ER via the SRP-dependent mechanism. The ADHAPR mutant lacking the N-terminal domain preferentially targets to the ER, restoring the reduced level of PlsEtn synthesis in FAA.K1B cell. In contrast, the expression of full-length ADHAPR in the mutant cells elevates the synthesis of phosphatidylethanolamine, but not PlsEtn. Taken together, these results suggest that the third step of plasmalogen synthesis is mediated by ER-localized ADHAPR.

## Introduction

Plasmalogen is a major class of glycerophospholipid containing a characteristic vinyl-ether bond at the *sn*-1 position of the glycerol backbone. Plasmalogens account for about 20% of total phospholipids in humans (Nagan and Zoeller, 2001). Ethanolamine plasmalogens (PlsEtns) are major constituents of biological membranes in most human tissues where they constitute approximately 5–20% of the phospholipids, while choline plasmalogens are major constituents primarily of cardiac tissue and skeletal muscle membranes (Braverman and Moser, 2012).

PlsEtns are synthesized in seven steps (Nagan and Zoeller, 2001). The initial two steps of plasmalogen biosynthesis in peroxisomes are well characterized. The first-step synthesis of plasmalogens is catalyzed by dihydroxyacetonephosphate acyltransferase/glyceronephosphate *O*-acyltransferase (DHAPAT/GNPAT; hereafter called DHAPAT), an intraperoxisomal protein facing the matrix side of the peroxisomal membrane, to generate *sn*-1-acyl-DHAP (acyl-DHAP/1-acyl-glyceron 3-phosphate; hereafter called acyl-DHAP) (Thai et al., 1997). In the next step, alkyl-DHAP synthase/alkylglycerone phosphate synthase (ADAPS/AGPS; hereafter called ADAPS) substitutes the acyl chain of acyl-DHAP to a long chain fatty alcohol to synthesize 1-*O*-alkyl-glycerone 3-phosphate (alkyl-DHAP) (Nagan and Zoeller, 2001). At the third step of plasmalogen synthesis, alkyl-DHAP is enzymatically reduced by acyl/alkyl DHAP reductase (ADHAPR), activity which is found in both peroxisomal and microsomal fractions in guinea pig liver (LaBelle and Hajra, 1974). The remaining four steps of plasmalogen synthesis are catalyzed by enzymes localized in the endoplasmic reticulum (ER), including TMEM189, an integral ER membrane protein catalyzing the formation of the vinyl-ether bond in the final step of plasmalogen synthesis (Gallego-García et al., 2019; Werner et al., 2020).

The facts that ADHAPR activities in peroxisomes and ER are simultaneously inhibited by thermal denaturation, NADP+, and acyl-CoA, and the isolation of a cell line, FAA.K1B, showing 95% reduction in the ADHAPR activity from mutagenized Chinese hamster ovary (CHO) cells, suggest that the identical protein is localized to both peroxisomes and ER (Ghosh and Hajra, 1986; James and Zoeller, 1997). Interestingly, only a moderate decrease in plasmalogen synthesis is detectable in the FAA.K1B cells, despite a severe reduction in ADHAPR activity, suggesting a shunt pathway that bypasses the step catalyzed by ADHAPR (James and Zoeller, 1997). A similar result of a moderate level of alkyl ether-glycerophosphocholine was reported in mouse embryonic cells by knocking down *DHRS7b*, a gene identified as a mammalian ortholog of yeast enzyme Ayr1p (EC:1.1.1.101) which catalyzes the reduction of acyl-DHAP (Lodhi et al., 2012). However, they did not examine the effects on the levels of PlsEtn. This loss of function study of *DHRS7b* suggests that *DHRS7b* gene encodes the mammalian ADHAPR, referred to as peroxisomal reductase-activating PPARγ (PexRAP) by the authors based on their finding that alkyl ether-glycerophosphocholine is associated with PPARγ and elevates the PPARγ transcriptional activity (Lodhi et al., 2012).

In the present study, we investigated whether the loss of function of ADHAPR reduces the synthesis of PlsEtn, the most abundant plasmalogen in mammalian tissues and culture cells. We also assessed the intracellular localization, membrane topology, and mechanism for organelle targeting of ADHAPR. Further analyses by the expression of ADHAPR and nucleotide-sequencing of *DHRS7b*-coding region in FAA.K1B cells revealed that ADHAPR encoded by *DHRS7b* indeed catalyzes the reduction of alkyl-DHAP in the ER, not in peroxisomes.

## Results

### Knockdown of *DHRS7b* Reduces Synthesis of PlsEtns

**FIGURE 1:**
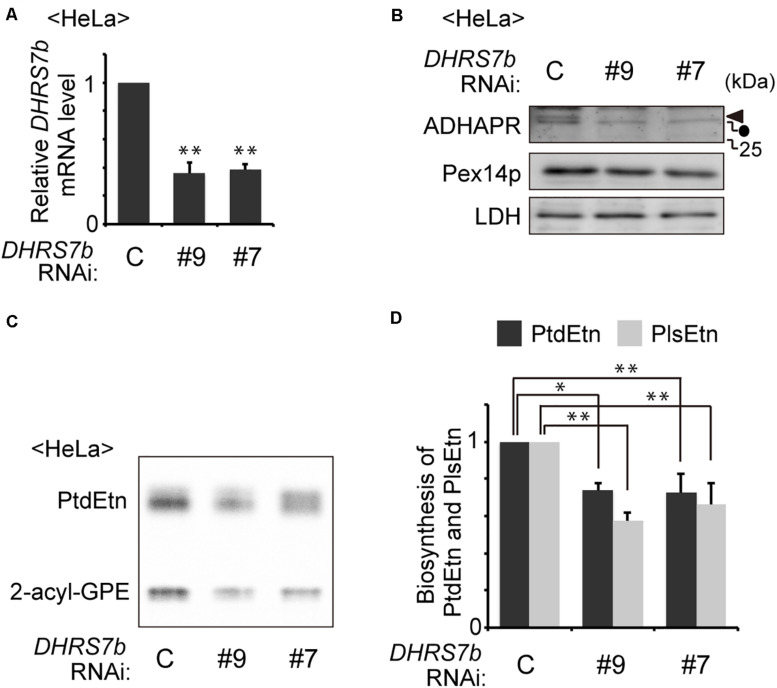
Knockdown of *DHRS7B* reduces biosynthesis of PlsEtn. **(A)** HeLa cells were transfected with two different dsRNAs (#9 and #7) against *DHRS7B* encoding ADHAPR and cultured for 72 h. Transcription level of *DHRS7B* was analyzed by quantitative real-time PCR. **(B)** Protein level of ADHAPR was analyzed by immunoblotting with the indicated antibodies. Lactate dehydrogenase (LDH), a cytosolic protein; Pex14p, a peroxisomal membrane protein (PMP). Solid arrowhead and dot indicate ADHAPR and a non-specific band, respectively. **(C)** HeLa cells cultured as described in A) were metabolically labeled for 2 h with ^14^C-Etn and assessed for the biosynthesis of PtdEtn and PlsEtn by converting PlsEtn to 2-acyl-GPE with trichloroacetic acid (Honsho et al., 2008). **(D)** Biosynthesis of PlsEtn and PtdEtn was represented as values relative to control HeLa cells. *p< 0.05, **p< 0.01; Student’s *t*-test versus control HeLa cells.

PlsEtns are found in various types of mammalian cells. Synthesis of PlsEtn is initiated in peroxisomes and completed in the ER via totally seven steps of reactions where alkyl-DHAP is reduced by the ADHAPR at the third step of seven-step reactions in the PlsEtn synthesis. Recent study showing the 40–50% reduced level of alkyl ether-glycerophosphocholine by the knockdown of *DHRS7b* (Lodhi et al., 2012), suggests that the enzyme encoded by *DHRS7b* likely acts as a reductase for alkyl-DHAP. Therefore, we tested whether synthesis of PlsEtn is inhibited by the knockdown of *DHRS7b*. Transfection of two independent siRNAs against *DHRS7b* in HeLa cells reduces transcription of *DHRS7b* by nearly 60% of that in mock-treated HeLa cells (Figure 1A), where the protein level of ADHAPR was reduced to an undetectable level, as assessed by immunoblotting with ADHAPR antibody (Figure 1B). Upon transfecting siRNA against *DHRS7b*, synthesis of PlsEtn and phosphatidylethanolamine (PtdEtn) was reduced about 40% of those in mock-treated cells (Figures 1C,D). Such reduced synthesis of PlsEtn and PtdEtn is also observed in the CHO cell mutant FAA.K1B in a short metabolic-labeling period with [1-^3^H]ethanolamine (Etn) due to the absence of ADHAPR activity (James and Zoeller, 1997). Together, these results suggest that the enzyme, ADHAPR, encoded by *DHRS7b* most likely catalyzes the reduction of acyl/alkyl-DHAP in plasmalogen biosynthesis.

### Intracellular Localization of the Protein Encoded by *DHRS7b*

**FIGURE 2:**
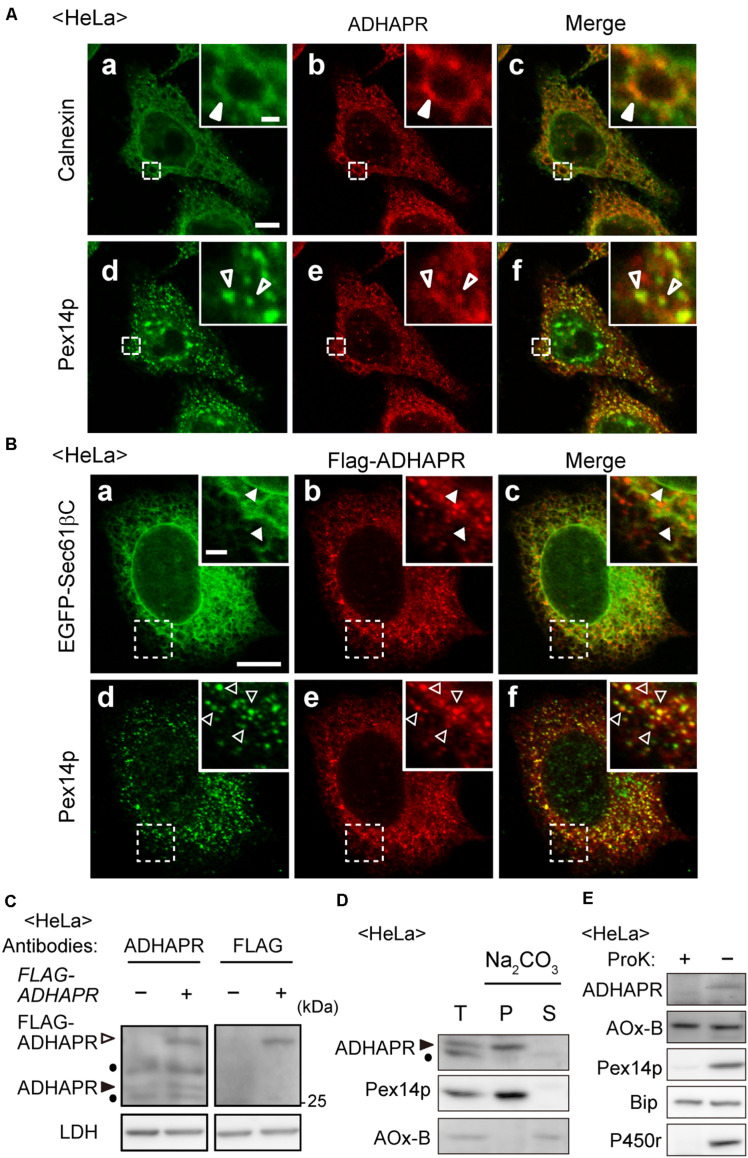
ADHAPR is localized to peroxisomes and ER. **(A)** Intracellular localization of endogenous ADHAPR was analyzed in HeLa cells by permeabilizing cells with semi-intact buffer prior to the fixation and subjected to the immunofluorescence staining using antibodies to ADHAPR (b and e). ER and peroxisomes were visualized by immunostaining with antibodies to calnexin (a) and Pex14p (d), respectively. Merged views of a with b and d with e are shown in c and f, respectively. Insets show the magnified images of the boxed areas. Solid and open arrowheads indicate colocalization of ADHAPR with calnexin and Pex14p, respectively. Bar, 10 and 2 μm (insets). **(B)** FLAG-ADHAPR was transiently expressed with the carboxy-terminal 25-amino acid residues of Sec61β fused to EGFP (EGFP-Sec61βC) for 24 h in HeLa cells and subjected to the immunofluorescence staining using antibodies to FLAG (b and e). ER and peroxisomes were visualized by EGFP-Sec61βC (a) and immunostaining with anti-Pex14p antibody (d), respectively. Merged views of a with b and d with e are shown in c and f, respectively. Insets show the magnified images of the boxed areas. Solid and open arrowheads indicate colocalization of FLAG-ADHAPR with EGFP-Sec61βC and Pex14p, respectively. Bar, 10 and 2 μm (insets). **(C)** FLAG-ADHAPR was expressed (+) as in **(B)** and its expression was assessed by the antibodies to ADHAPR and FLAG as indicated at the top. Open and solid arrowheads indicate FLAG-ADHAPR and endogenous ADHAPR, respectively. LDH was used as a loading control. Dots indicate a non-specific band. **(D)** Organelle fraction prepared from postnuclear supernatants (T) of HeLa cells were treated with 0.1 M Na_2_CO_3_ and separated into soluble (S) and membrane (P) fractions. Equal aliquots of respective fractions were analyzed by immunoblotting with the indicated antibodies. Acyl-CoA oxidase (AOx), a peroxisomal matrix enzyme. Of polypeptide chains of AOx (A, B, and C polypeptides), only the B chain is shown. Arrowhead indicates ADHAPR. Dot: a non-specific band. **(E)** Postnuclear supernatants (–) of HeLa cells were treated with 50 μg/ml proteinase K for 30 min on ice (+) and analyzed by immunoblotting with the indicated antibodies. BiP, an ER luminal chaperon binding to immunoglobulin; P450r, an ER membrane protein.

ADHAPR activity was found in peroxisomal and microsomal fractions in the liver of guinea pig and rat (LaBelle and Hajra, 1974; Ghosh and Hajra, 1986). The microsomal and peroxisomal ADHAPR show similar properties with respect to the pH optimum, heat stability, substrate specificity, and kinetic properties by which it is generally considered that the same enzyme is present in both peroxisomal and microsomal fractions (Ghosh and Hajra, 1986). Therefore, we investigated the intracellular localization of endogenous ADHAPR in HeLa cells by immunostaining with anti-ADHAPR antibody. As anticipated, ADHAPR co-localized with peroxisomal membrane protein peroxin 14 (Pex14p) and calnexin, an ER resident molecular chaperone in HeLa cells (Figure 2A). Similarly, N-terminally FLAG-tagged ADHAPR, FLAG-ADHAPR, co-localized with both Pex14p and EGFP-Sec61βC, an ER-localized GFP fusion protein containing the C-terminal transmembrane segment of ER marker protein Sec61β (Yagita et al., 2013; Figure 2B), implying that N-terminally tagged FLAG peptide does not interfere with the targeting of ADHAPR to peroxisomes and ER. Under this condition, the expressed FLAG-ADHAPR was detected as a slower-migrating band with both ADHAPR- and FLAG-antibodies just above the endogenous ADHAPR (Figure 2C). Taken together, these results suggest that ADHAPR is localized to both organelles, peroxisomes and ER.

We further assessed the membrane topology of ADHAPR. On the basis of its primary sequence, ADHAPR is predicted to possess a single putative transmembrane domain (TMD) at its N-terminal region. Indeed, endogenous ADHAPR was found to be in the membrane fraction and resistant to the alkaline extraction, similar to Pex14p, an integral membrane protein resides in peroxisomes (Figure 2D), indicating that ADHAPR is localized as an integral membrane protein. Moreover, ADHAPR was not detected with the antibodies recognizing the central portion of ADHAPR upon treating organelle fractions with proteinase K (Figure 2E), suggesting that ADHAPR is localized to peroxisomes and ER and exposes its catalytic C-terminal domain to the cytosol.

**FIGURE 3:**
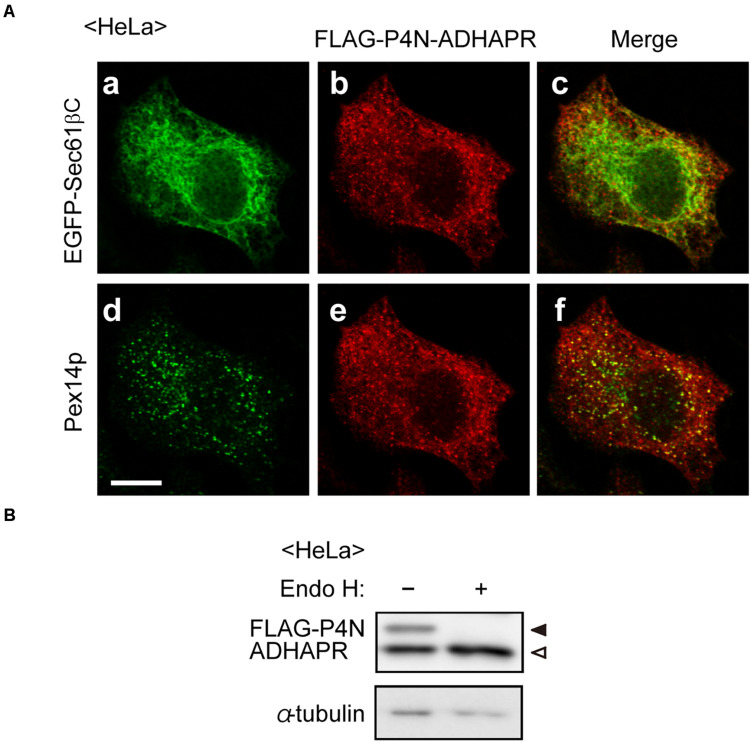
Assessment of membrane topology of ADHAPR in the ER. **(A)** HeLa cells expressing FLAG-P4N-ADHAPR, ADHAPR mutant substituted at proline 4 with asparagine to create N-linked consensus sequences, and EGFP-Sec61βC for 24 h were analyzed as in Figure 2B. Merged views of a with b and d with e are shown in c and f, respectively. Bar, 10 μm. **(B)** Whole cell lysates (–) of HeLa cells expressing FLAG-P4N-ADHAPR for 24 h were treated with endoglycosidase H (Endo H, +) and analyzed by immunoblotting with antibodies to FLAG (upper panel) and α-tubulin (lower panel). Solid and open arrowheads indicate glycosylated and non-glycosylated FLAG-P4N-ADHAPR, respectively. α-tubulin was used as a loading control.

The topology of N-terminal domain of ADHAPR in the ER was further assessed by the post-translational modification with N-glycosylation to P4N-ADHAPR, the ADHAPR mutant substituting the proline at position 4 of ADHAPR to asparagine to generate the N-linked glycosylation consensus sequence. Immunofluorescence microscopic analysis showed that FLAG-P4N-ADHAPR was localized to both peroxisomes and the ER, similar to the endogenous ADHAPR (Figure 3A). FLAG-P4N-ADHAPR was detected as double bands with FLAG-antibody, in which the slower migrating band disappeared upon endoglycosidase H treatment (Figure 3B), hence implying that the N-terminal FLAG-P4N-ADHAPR penetrated into the ER lumen. Collectively, these results suggest that ADHAPR is most likely localized in peroxisomes and the ER as a type I integral membrane protein.

### ADHAPR Targets to Peroxisomes via a Pex19p-Dependent Class I Pathway

Several types of peroxisomal membrane proteins (PMPs), including tail-anchored and multi-spanning PMPs, are delivered to peroxisomes in a Pex19p- and Pex3p-dependent class I pathway. In this pathway, Pex19p, a predominantly cytosolic protein, forms a complex with newly synthesized PMPs in the cytosol, delivers it to the membrane receptor Pex3p on peroxisomes (Jones et al., 2004; Yagita et al., 2013). Moreover, recent study showed that ATAD1 (ATPase family AAA domain-containing protein 1), N-terminally anchored protein, is localized to peroxisomes in a manner dependent on the class I pathway and mitochondria (Liu et al., 2016). Since ADHAPR is a type I integral membrane protein localized in peroxisomes and the ER, we examined whether ADHAPR targets to peroxisomes via the Pex19p-dependent class I pathway as ATAD1.

**FIGURE 4:**
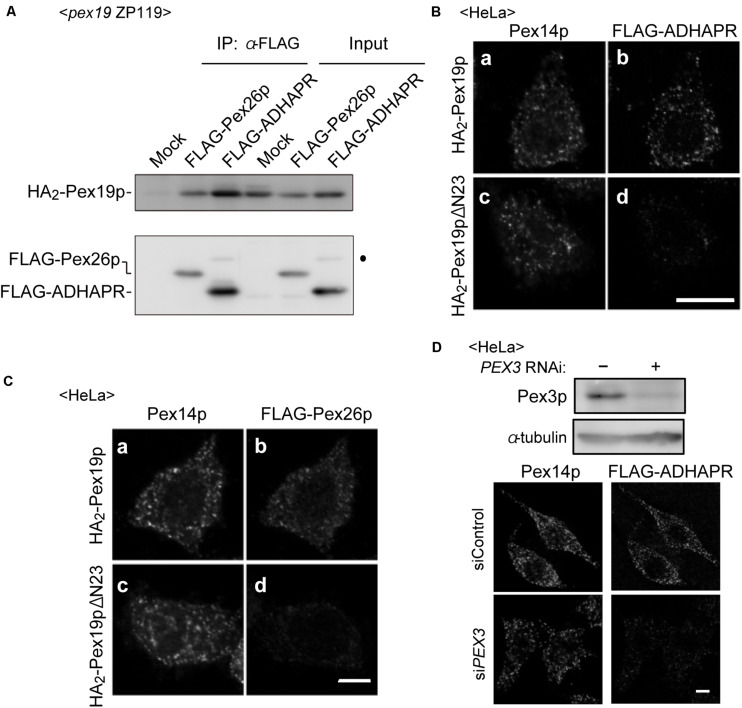
ADHAPR targets to peroxisomes by a Pex19p-dependent class I pathway. **(A)** Cytosolic fractions of *PEX19*-defective *pex19* CHO ZP119 cells co-expressing HA_2_-Pex19p and either FLAG-ADHARP or FLAG-Pex26p were subjected to immunoprecipitation with anti-FLAG agarose beads. Immunoprecipitates were analyzed by immunoblotting using antibodies to HA (upper panel) and FLAG (lower panel), respectively. Dot indicates a non-specific band. **(B,C)** FLAG-ADHAPR **(B)** and FLAG-Pex26p **(C)** were synthesized in a rabbit reticulocyte lysate (RRL) translation system supplemented with HA_2_-Pex19p (a and b) or HA_2_-Pex19pΔN23 (c and d) synthesized in RRL, and further incubated at 26°C for 1 h with semi-intact HeLa cells. HA_2_-Pex19pΔN23 is a Pex19p variant lacking the N-terminal 23 amino acid residues required for the binding to Pex3p. The cells were subjected to the immunofluorescence staining. FLAG-ADHAPR and FLAG-Pex26p were detected with anti-FLAG antibody (b and d), and peroxisomes were visualized by immunostaining with anti-Pex14p antibody (a and c), respectively. Bar, 10 μm. **(D)** Targeting of FLAG-ADHAPR was assessed in HeLa cells transfected with control dsRNA (–) or dsRNA against *PEX3* as in **(B)**. α-tubulin was used as a loading control. Bar, 10 μm.

Co-immunoprecipitation study revealed that co-expression of FLAG-ADHAPR together with N-terminally 2 × HA-tagged Pex19p (HA_2_-Pex19p) gave rise to the formation of cytosolic FLAG-ADHAPR-HA_2_-Pex19p complexes similar to the formation of a complex between Pex19p and Pex26p, a C-tail anchored membrane peroxin (Matsumoto et al., 2003; Figure 4A). To examine whether the ADHAPR-Pex19p complex is an import-competent intermediate, a targeting assay was performed *in vitro* using semi-intact cells (Matsuzaki and Fujiki, 2008). HeLa cells were treated with digitonin to selectively permeabilize the plasma membrane. FLAG-ADHAPR was synthesized in a rabbit reticulocyte lysate (RRL) translation system supplemented with RRL-synthesized HA_2_-Pex19p or HA_2_-Pex19pΔN23 and then overlaid on semi-permeabilized HeLa cells. HA_2_-Pex19pΔN23 is a Pex19p mutant lacking the N-terminal 23 amino acid residues, an essential domain for binding to the membrane receptor Pex3p, thereby defective in binding to Pex3p (Matsuzono et al., 2006; Yagita et al., 2013). Immunofluorescence microscopy analysis showed that FLAG-ADHAPR and FLAG-Pex26p both synthesized in the presence of HA_2_-Pex19p, coincided with Pex14p (Figures 4B,C), indicating that FLAG-ADHAPR was targeted to peroxisomes. In contrast, HA_2_-Pex19pΔN23 failed to deliver both cargo proteins to peroxisomes (Figures 4B,C). Furthermore, the subsequent *in vitro* import assay using Pex3p-depleted semi-permeabilized HeLa cells showed that the peroxisomal targeting of FLAG-ADHAPR was severely abrogated (Figure 4D), hence indicating that the peroxisomal targeting of ADHAPR requires the membrane receptor Pex3p. Altogether, these results suggest that ADHAPR targets to peroxisomes via a Pex19p- and Pex3p-dependent class I pathway.

### ADHAPR Directly Targets to the ER in a Pex19p- and Pex3p-Independent Manner

**FIGURE 5:**
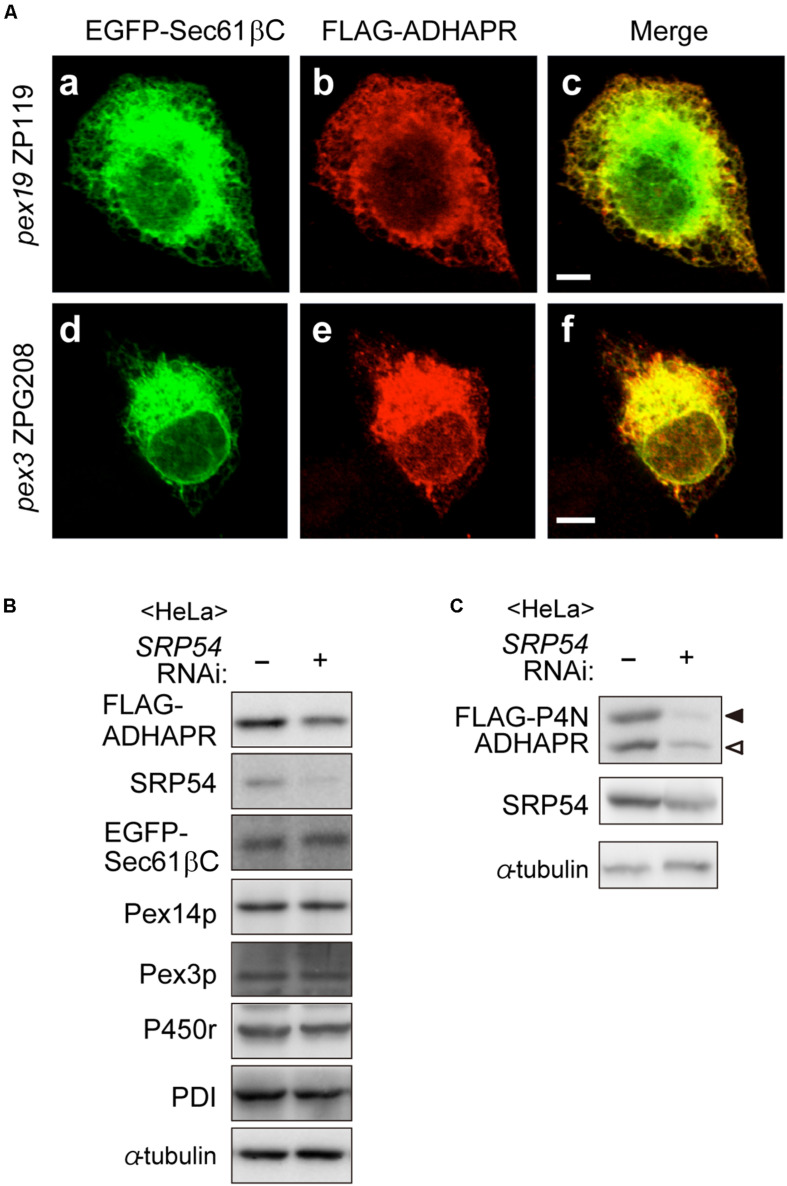
ADHAPR targets to the ER in a Pex19p and Pex3p-independent manner. **(A)** EGFP-Sec61βC and FLAG-ADHAPR were expressed in *PEX19*-defective *pex19* ZP119 cells and *PEX3*-defective *pex3* CHO ZPG208 cells. The cells were cultured for 48 h and subjected to the immunofluorescence staining. FLAG-ADHAPR was detected with anti-FLAG antibody (b and e). ER was visualized by EGFP-Sec61βC (a and d). Bars, 5 μm. **(B)** HeLa cells were transfected with siRNA against *SRP54* for 66 h and then transfected with FLAG-ADHAPR together with EGFP-Sec61βC, followed by analyzing the level of proteins using the indicated antibodies. SRP54, the 54 kDa subunit of the signal recognition particle; Pex3p, a PMP; PDI (protein disulfide isomerase), an ER luminal protein. **(C)** Protein level of glycosylated- (solid arrowhead) and non-glycosylated (open arrowhead)-FLAG-P4N-ADHAPR were verified as in **(B)**. α-tubulin, a loading control.

Although ADHAPR targets to peroxisomes via a Pex19p-Pex3p-dependent class I pathway (Figure 4), mechanisms for targeting to the ER remain unclear. Interestingly, UbxD8, a subfamily of hair pin proteins localizing to the ER and lipid droplets targets to the ER via a Pex3p-Pex19p-dependent mechanisms (Schrul and Kopito, 2016). Therefore, we investigated whether ADHAPR also targets to the ER by Pex19p- and Pex3p-dependent pathway. To this end, FLAG-ADHAPR was expressed in two CHO mutant cell lines; *PEX19*-defective *pex19* ZP119 cells and *PEX3*-defective *pex3* ZPG208 (Figure 5A; Kinoshita et al., 1998; Ghaedi et al., 1999). FLAG-ADHAPR was localized to the ER in *pex19* ZP119 and *pex3* ZPG208 cells, both lacking peroxisome membrane due to the absence of Pex19p and Pex3p expression (Kinoshita et al., 1998; Ghaedi et al., 1999; Matsuzono et al., 1999). These results suggest that ADHAPR targets to the ER in a manner independent of Pex19p- and Pex3p.

To elucidate the ER targeting pathway of ADHAPR, we next focused on the signal recognition particle (SRP) pathway, a well-studied ER targeting mechanism mediated by a N-terminal hydrophobic signal sequence. ADHAPR resides in the ER as a type I integral membrane protein by penetrating its N-terminal domain through a single putative TMD in its N-terminal region (Figure 2), thus raising the possibility that ADHAPR is co-translationally inserted to the ER by the SRP pathway. It has been observed that knockdown of SRP causes a reduced level of preprolactin, a cotranslationally targeted secretory protein (Karamyshev et al., 2014). Therefore, we analyzed the protein level of several membrane proteins including the expressed FLAG-ADHAPR and EGFP-Sec61βC in HeLa cells transfected with siRNA against *SRP54* (Kanda et al., 2016). In SRP54-depleted HeLa cells, the protein level of FLAG-ADHAPR was less than that in mock-treated HeLa cells (Figure 5B). Similarly, less amount of the oligosaccharide modified and unmodified FLAG-P4N-ADHAPR was observed by reduced SRP54 expression in HeLa cells (Figure 5C). Contrary to this, protein level of EGFP-Sec61βC was not altered by the reduction of SRP54 (Figure 5B). Collectively, ADHAPR is most likely inserted into the ER via the SRP-dependent mechanism.

### The Role of ADHAPR in Peroxisomes and ER

**FIGURE 6:**
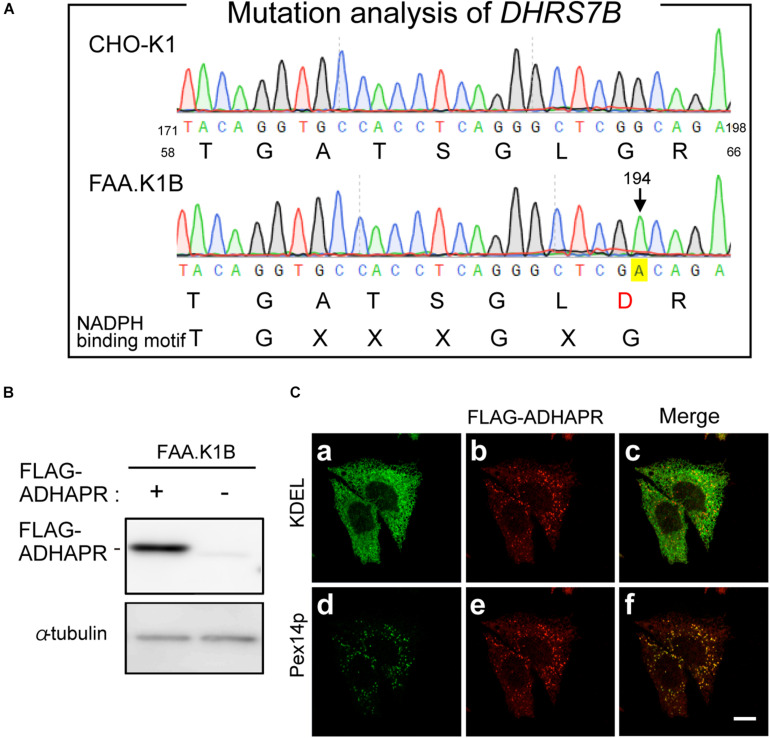
Mutation analysis of FAA.K1B and isolation of FAA.K1B stably expressing FLAG-ADHAPR. **(A)** Mutation analysis of *DHRS7B* from FAA.K1B, a mutant CHO cell line defective in ADHAPR. Partial nucleotide sequence and deduced amino acid sequence of DHRS7B cDNA isolated from a wild-type (upper panel) and FAA.K1B (lower panel) are shown. A point mutation at nucleotide position 194 in *DHRS7B* caused missense mutation from G to A in a codon for Gly at amino acid position 65 in ADHAPR in FAA.K1B cells is marked with yellow box. The consensus sequence for NADPH-binding is represented at the bottom. **(B)** Protein level of FLAG-ADHAPR in a FAA.K1B cell line expressing FLAG-ADHAPR (+). FLAG-ADHAPR was detected by monoclonal anti-FLAG antibody. α-tubulin, a loading control. **(C)** Intracellular localization of FLAG-ADHAPR was assessed in the FAA.K1B cell line stably expressing FLAG-ADHAPR by the immunofluorescence staining. FLAG-ADHAPR was verified with anti-FLAG antibody (b and e). ER and peroxisomes were visualized by immunostaining with antibodies to KDEL (Lys-Asp-Glu-Leu), the most common ER retention signal (a) and Pex14p (d), respectively. Merged views of a with b and d with e are shown in c and f, respectively. Bar, 10 μm.

To gain further insight of dual localization of ADHAPR, we focused on the CHO mutant cell, FAA.K1B, with a deficiency in ADHAPR activity (James et al., 1997). The severely reduced activity of ADHAPR in FAA.K1B was shown using either acyl-DHAP or alkyl-DHAP as a substrate (James et al., 1997). These studies together with our observations in terms of intracellular localization of ADHAPR suggest that dysfunction of ADHAPR activity is caused by a mutation in the *DHRS7B* in FAA.K1B cells. By amplifying partial DNA fragments of *DHRS7B* derived from FAA.K1B and CHO-K1 cells, a roughly equal amount of DNA fragments was obtained using the first strand cDNA from respective cells with three different sets of primers targeting to distinct regions of ADHAPR coding sequence (data not shown). We next conducted mutation analysis of *DHRS7B* in FAA.K1B cells and identified a missense mutation from G to A at nucleotide position 194 in a codon for Gly to Asp in *DHRS7B* in FAA.K1B cells (Figure 6A). This missense mutation is located on the consensus sequence for NADPH-binding (TGxxxGxG) (Jörnvall et al., 1995; Filling et al., 2002), thereby suggesting that an impaired NADPH binding to ADHAPR diminished the activity of ADHAPR in FAA.K1B cells.

**FIGURE 7:**
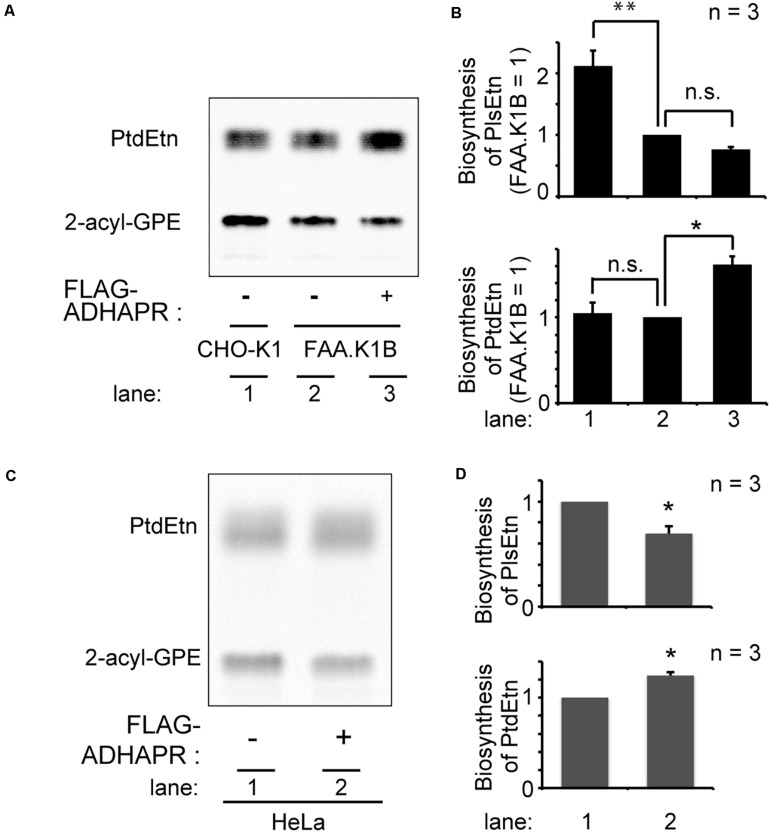
Expression FLAG-ADHAPR elevates the synthesis of PtdEtn but not PlsEtn. **(A)** Synthesis of PtdEtn and PlsEtn was verified in CHO-K1 (lane 1), FAA.K1B (lane 2), and a FAA.K1B cell lines stably expressing FLAG-ADHAPR (lane 3) as in Figure 1C. **(B)** Biosynthesis of PlsEtn (upper panel) and PtdEtn (lower panel) was represented by taking as one that in FAA.K1B cells. **p< 0.01, *p< 0.05, *t*-test versus control FAA.K1B cells. n.s., not significant; one-way ANOVA with Dunnett’s *post hoc* test as compared with FAA.K1B cells. **(C)** Synthesis of PtdEtn and PlsEtn was assessed in HeLa cells transiently expressing mock (–) or FLAG-ADHAPR (+) as in **(A)**. **(D)** Biosynthesis of PlsEtn (upper panel) and PtdEtn (lower panel) was represented by taking as one that in mock transfected (–) HeLa cells. *p < 0.05, *t*-test versus HeLa cells.

Next, we attempted to restore the synthesis of PlsEtn and PtdEtn in FAA.K1B cell lines by stably expressing FLAG-ADHAPR. By selecting cells expressing FLAG-ADHAPR with Zeocin (Honsho et al., 2013), a cell line, termed FAA.K1B/FLAG-ADHAPR was isolated (Figure 6B). FLAG-ADHAPR was localized to peroxisomes and ER (Figure 6C). Subsequently, we analyzed lipid synthesis in the isolated cells by metabolically labeling with ^14^C-Etn. We unexpectedly found that the reduced level of the PlsEtn synthesis in FAA.K1B cells was not restored in the cells stably expressing FLAG-ADHAPR (Figures 7A,B). Moreover, synthesis of PtdEtn was dramatically increased as compared to that in FAA.K1B cells. Essentially, the same results were obtained upon expressing FLAG-ADHAPR in HeLa cells (Figures 7C,D). Taken together, these results suggest that ADHAPR enhances the synthesis of non-ether glycerophospholipids, at least PtdEtn in both cell lines. We therefore interpreted these results to mean that ADHAPR preferentially reduces acyl-DHAP prior to the synthesis of alkyl-DHAP on peroxisomes.

**FIGURE 8:**
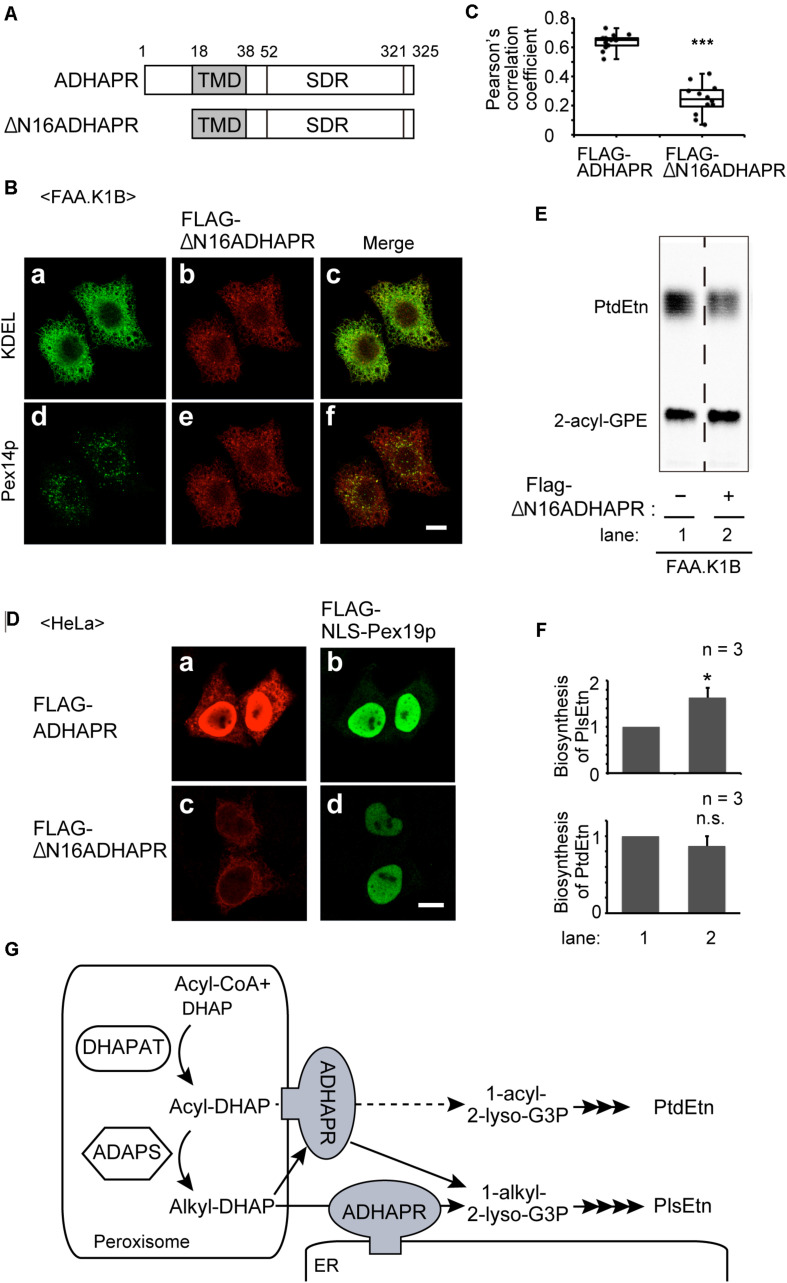
The ER-localized ADHAPR elevates PlsEtn synthesis in FAA.K1B cells. **(A)** Schematic representation of ADHAPR variants used. FLAG-ΔN16ADHAPR was lacking N-terminal 16-amino acids of ADHAPR. Numbers, amino acid residues of human ADHAPR; solid box, transmembrane domain (TMD); SDR, short-chain dehydrogenase/reductase domain. **(B)** FLAG-ΔN16ADHAPR was stably expressed in FAA.K1B cells. FLAG-ΔN16ADHAPR was detected with anti-FLAG antibody (b and e). ER and peroxisomes were visualized by immunostaining with antibodies to KDEL (a) and Pex14p (d), respectively. Merged views of a with b and d with e are shown in c and f, respectively. Scale bar, 10 μm. **(C)** Typical immunofluorescent images of FAA.K1B cells stably expressing FLAG-ADHAPR and FLAG-ΔN16ADHAPR are shown in Supplementary Figure S1. Pearson’s correlation coefficient for colocalization of Pex14p with FLAG-ADHAPR (*n* = 14) and FLAG-ΔN16ADHAPR (*n* = 12) was determined. ***p < 0.001 analyzed by Mann-Whitney *U*-test. **(D)** *FLAG-NLS-PEX19* was expressed for 7 h together with *FLAG-ADHAPR* (a and b) or *FLAG-ΔN16ADHAPR* (c and d) in HeLa cells. FLAG-ADHAPR and FLAG-ΔN16ADHAPR were detected with anti-ADHAPR antibody (a and c). FLAG-NLS-Pex19p was detected with anti-Pex19p (b and d). Bar, 10 μm. **(E)** Synthesis of PtdEtn and PlsEtn was assessed in FAA.K1B (lane 1) and FLAG-ΔN16ADHAPR-expressing FAA.K1B (lane 2). **(F)** By quantifying the data shown in **(E)**, biosynthesis of PlsEtn (upper panel) and PtdEtn (lower panel) was represented by taking as one that in FAA.K1B cells. *p < 0.05, *t*-test versus control FAA.K1B cells. **(G)** A schematic model of roles of ADHAPR in the ER and peroxisomes. ER-localized ADHAPR catalyzes a reduction of alkyl-DHAP, while ADHAPR on peroxisomes prefers to reduce acyl-DHAP to synthesize 1-acyl-2-lyso-G3P (dashed line), a product generated by the action of DHAPAT, prior to a subsequent synthesis of alkyl-DHAP, although it can also catalyze the reduction of alkyl-DHAP.

To further assess the functional difference of ADHAPRbetween in peroxisomes and ER, we attempted to generate ADHAPRpreferentially localizing in the ER. We showed that ADHAPR was co-translationally targeted to the ER in an SRP-dependent manner (Figure 5). Nevertheless, much ADHAPR targets to peroxisomes in cells as judged by the immunofluorescence pattern of cells expressing ADHAPR (Figures 2A,B). From these results, we suspected that Pex19p directly binds to its N-terminal region of the nascent ADHAPR prior to the recognition by SRP and delivers it to peroxisomes. We, therefore, expressed FLAG-ΔN16ADHAPR lacking N-terminal 16-amino acids of ADHAPR (Figure 8A) and assessed its intracellular localization in FAA.K1B cells (Figure 8B) as in Figure 2. FLAG-ΔN16ADHAPR was predominantly localized to the ER, while peroxisomal localization was markedly reduced (Figure 8B) as compared to the localization of FLAG-ADHAPR (Figures 6C, 8C). Expression of Pex19p tagged with nuclear localization signal (FLAG-NLS-Pex19p) strongly interfered FLAG-ADHAPR from peroxisomal localization, implying that FLAG-NLS-Pex19p transported the newly synthesized FLAG-ADHAPR to the nucleus (Figure 8D). In contrast, FLAG-NLS-Pex19p did not alter the ER localization of FLAG-ADHAPR (Figure 8D). However, FLAG-ΔN16ADHAPR was not discernible in the nucleus, even in the coexpression with FLAG-NLS-Pex19p (Figure 8D). Together, these results suggest that Pex19p recognizes the N-terminal of ADHAPR on free ribosomes, possibly prior to exposing its hydrophobic TMD, a putative SRP-binding domain of ADHAPR. Interestingly, synthesis of PlsEtn but not PtdEtn, was highly elevated in FAA.K1B/FLAG-ΔN16ADHAPR cells (Figures 8E,F). Collectively, these results suggest that ADHAPR reduces alkyl-DHAP rather than acyl-DHAP in the ER, while ADHAPR in peroxisomes prefers to catalyze the reduction of acyl-DHAP, a product generated by the action of DHAPAT, prior to a subsequent synthesis of alkyl-DHAP catalyzed by ADAPS (Figure 8G).

## Discussion

In the present study, we show that ADHAPR encoded by *DHRS7B* targets to peroxisomes and ER, which is consistent with the earlier findings that ADHAPR activities are found in both peroxisomal and microsomal fractions in rodents (LaBelle and Hajra, 1974; Ghosh and Hajra, 1986). We also show that synthesis of PtdEtn and PlsEtn is impaired in *ADHAPR*-knocked down HeLa cells, essentially the same as in the mutant cell FAA.K1B, a CHO cell line defective in acyl/alkyl-DHAP reductase activity (James et al., 1997). Identification of missense mutation in NADPH-binding domain of ADHAPR from FAA.K1B confirmed that ADHAPR is the enzyme responsible for catalyzing the third step of plasmalogen biosynthesis.

Using a digitonin-permeabilized cell-based import assay system, we showed that ADHAPR is targeted to peroxisomes in a Pex19p-dependent Class I pathway. ATAD1, an N-terminally anchored protein localizing peroxisomes and mitochondria, is likewise targeted to peroxisomes in a manner dependent on the Class I pathway (Liu et al., 2016). In contrast, ΔN16ADHAPR lacking the N-terminal hydrophilic region is predominantly localized to the ER and its peroxisomal localization is severely impaired (Figure 8). Furthermore, FLAG-ADHAPR, but not FLAG-ΔN16ADHAPR, is transported to the nucleus upon co-expressing with NLS-tagged Pex19p (Figure 8). These results suggest that Pex19p is able to bind to the nascent ADHAPR on free ribosomes, possibly prior to exposing its hydrophobic transmembrane domain, followed by releasing into cytosol by forming a Pex19p-ADHAPR complex as an import-competent intermediate (Figure 4), as illustrated in the earlier study suggesting the mechanism underlying the peroxisome targeting of a multi-spanning membrane protein, PMP70 (Kashiwayama et al., 2007). Recent study showed that the short segment composed of nine residues in the N-terminal of PMP70 acts as a suppressor for cotranslational targeting of PMP70 to the ER, where serine at amino-acid position 5 is essential (Sakaue et al., 2016). However, the amino acid sequence of the N-terminal hydrophilic region of ADHAPR is dissimilar to that in PMP70, suggesting that any potential proteins recognizing the N-terminal short segment of PMP70 is unlikely involved in the transport of ADHAPR to peroxisomes.

We found that ADHAPR is localized to the ER in *pex19* and *pex3* mutant CHO cells devoid of peroxisomal membrane and lacking Pex19p (Kinoshita et al., 1998; Matsuzono et al., 1999) or Pex3p (Ghaedi et al., 1999; Figure 5A), hence implying that ADHAPR is targeted to the ER, not via peroxisomes and not involving Pex19p or Pex3p. These results are distinct from the Pex19p- and Pex3p-dependent ER localization of UbxD8 (Schrul and Kopito, 2016). Besides this peroxin-mediated ER targeting mechanism, three pathways are reported for the targeting mechanisms of membrane proteins to the ER including SRP-mediated co-translational pathway (Walter and Johnson, 1994), TRC40-mediated post-translational pathway (GET pathway) (Borgese and Fasana, 2011; Hegde and Keenan, 2011), and SRP-independent proteins-mediated targeting pathway (Aviram et al., 2016; Haßdenteufel et al., 2017). ADHAPR contains the hydrophobic TMD in the N-terminal region, thereby it is unlikely that ADHAPR is delivered to the ER via GET- or SRP-independent targeting-proteins-mediated ER targeting mechanism. Kyte and Doolittle plots with a window set of 15 amino acids give a hydrophobicity score 2.39 in TMD of ADHAPR, which is slightly higher than the hydrophobic score (2.29) of NADPH cytochrome P450r, suggesting that ADHAPR is localized to the ER in a manner dependent on SRP, similar to P450r (Neve and Ingelman-Sundberg, 2008). Consistent with this notion, knockdown of *SRP54* reduced the protein levels of the expressed FLAG-ADHAPR and N-glycosylated FLAG-P4N-ADHAPR (Figures 5B,C). Therefore, it is more likely that N-terminal hydrophobic segment of ADHAPR is recognized by SRP and that ADHAPR is directly transported to the ER in a manner dependent on the SRP-mediated co-translational pathway. Given the finding that FLAG-ADHAPR is still targeted to the ER upon co-expressing with NLS-tagged Pex19p (Figure 8D), recognition of the nascent ADHAPR by Pex19p may not be efficient, possibly owing that ribosomes translate mRNAs at high speed (approximately 6 amino acids per second) in mammalian cells (Boström et al., 1986; Ingolia et al., 2011), under which ADHAPR is able to target to the ER in a manner dependent on SRP. Furthermore, the peroxisome targeting of ADHAPR may be mediated by redundant mechanisms. Because recruitment of Pex19 to the N-terminus of the nascent ADHAPR located at the ribosomal tunnel exit has a potential to delay the association of SRP with the SRP receptor, which causes the dissociation of SRP from ribosomes (Lee et al., 2018), thereby allowing the transport of ADHAPR via conventional class I pathway mediated by posttranslational binding of Pex19p to ADHAPR in the cytosol as shown in semi-intact import assay of FLAG-ADHAPR synthesized in the RRL translation system (Figures 4B,D).

Noteworthily, ADHAPR, an integral membrane protein, is localized to the nucleus in the cells such as 3T3-L1 adipocytes and the differentiated PPARγ-overexpressing mouse embryonic fibroblasts by interacting with importin-β1 through the internal nuclear localization signal located in the C-terminal portion of ADHAPR. It has been shown that ADHAPR disrupts the complex of PPARγ and PRDM16, a critical transcription factor for thermogenesis, in the nucleus by interacting with PPARγ and PRDM16, leading to inhibit PRDM16-mediated adipocyte browning and expression of thermogenic genes such as UCP-1 (Lodhi et al., 2017). Peroxisomal localization of ADHAPR in 3T3-L1 adipocytes is suggested from findings of a recovery of PexRAP in fractions containing a peroxisomal marker PMP70 prepared from 3T3-L1 adipocytes and the binding of PexRAP to Pex19p (Lodhi et al., 2012). Collectively, multi-organelle targeting of ADHAPR is likely to be regulated by several factors including Pex19p, SRP, and importin-β1 in adipocytes. However, we could not observe nuclear localization of ADHAPR in HeLa cells, suggesting that targeting of the newly synthesized ADHAPR to the nucleus in adipocytes is regulated by the recognition of the nascent ADHAPR by at least three distinct factors.

The functional difference of ADHAPR localized to either ER or peroxisomes, has not been addressed. Expression of FLAG-ΔN16ADHAPR preferentially elevates the synthesis of PlsEtn, but not PtdEtn (Figure 8). We interpret these results to mean that the ER-localized ADHAPR preferentially catalyzes the reduction of alkyl-DHAP generated by ADAPS in peroxisomes. In the present study, we were unable to address the contribution of peroxisome-localized ADHAPR in the reduction of alkyl-DHAP because of no available ADHAPR mutant localized specifically to peroxisomes. The report that ADHAPR is mostly localized to peroxisomes in differentiated 3T3-L1 adipocytes with a normal level of plasmalogens (Hajra et al., 2000; Lodhi et al., 2012) suggests that alkyl-DHAP is reduced in both peroxisomes and ER (Figure 8). However, ADHAPR appears to catalyze the reduction of acyl-DHAP when ADHAPR is highly expressed and localized to peroxisomes as shown by the elevation of the synthesis of PtdEtn and reduced level of plasmalogen synthesis in HeLa and FAA.K1B cells exogenously expressing FLAG-ADHAPR (Figure 7). Interestingly, activities of DHAPAT and ADHAPR, but not ADAPS, are increased several-fold by the elevation of mRNA of respective genes during differentiation of 3T3-L1 adipocyte precursor cells to adipocytes, allowing the synthesis of about a half of triacylglycerol through this acyl-DHAP pathway (Hajra et al., 2000) without lowering the plasmalogen synthesis. This is consistent with the fact that ADHAPR is enriched in peroxisome fractions in 3T3-L1 adipocytes (Lodhi et al., 2012). Collectively, these results imply that DHAPAT and ADHAPR coordinately provide acyl-glycerol-3-phosphate for the synthesis of non-ether glycerophospholipids in peroxisomes. Under such experimental conditions, peroxisomal localization of ADHAPR might be regulated by accelerating targeting of ADHAPR to peroxisomes and/or by suppressing ADHAPR degradation in peroxisomes. In this context, it is interesting that transcription of mRNAs encoding PMPs including Pex19p is acutely up-regulated in human skeletal muscle at 4 h post-supplementation of high fat meal (Huang et al., 2019). Moreover, ADHARP is highly expressed in several tissues including liver, white adipocytes, and brain (Lodhi et al., 2012), suggesting that acyl-DHAP pathway is involved in the synthesis of non-ether glycerophospholipids. Therefore, intracellular localization of ADHARP appears to be regulated by its expressed level. Alternatively, the altered affinity of ADHAPR to Pex19p might modulate the intracellular localization of ADHAPR. Further studies addressing how the glycerolipid synthesis is accommodated by modulating the localization of ADHAPR to respond the environmental stimuli are required under physiological conditions.

## Materials and Methods

### Cell Culture, DNA Transfection, and RNAi

CHO cells, including CHO-K1, CHO *pex19* ZP119 (Kinoshita et al., 1998), CHO *pex3* ZPG208 (Ghaedi et al., 2000), and FAA.K1B (James et al., 1997), were maintained in Ham’s F-12 medium (Invitrogen) supplemented with 10% FBS (Biowest). HeLa cells were maintained in DMEM (Invitrogen) supplemented with 10% FBS (Biowest). All cell lines were cultured at 37°C under 5% CO_2_. DNA transfections were performed using Lipofectamine 2000 (Invitrogen) for HeLa cells, and Lipofectamine reagent (Invitrogen) for CHO cells according to the manufacture’s instructions and cells were cultured for the indicated time periods.

si*RNA*-mediated knockdown of *DHRS7B* and *PEX3* in HeLa cells was performed using predesigned Stealth^TM^ siRNAs (Invitrogen) using Lipofectamine 2000 and harvested at 72 h. The following siRNAs were used. Human*DHRS7B* #7 sense: 5′-AUACUGUUCCAUCUCGGCACGCAGA-3′ antisense: 5′-U CUGCGUGCCGAGAUGGAACAGUAU-3′, human*DHRS7B* #9 sense: 5′-UAACUCCAUACCUAGAUCCAUCCGC-3′antisense: 5′-GCGGAUGGAUCUAGGUAUGGAGUUA-3′, human*PEX3* sense: 5′-UAUUUACCUGGAUAAUGCAGCAGUU-3′antisense: 5′-AACUGCUGCAUUAUCCAGGUAAAUA-3′.

Knockdown of *SRP54* was likewise performed by transfecting the dsRNAs for human SRP54. The target sequence of the dsRNA is as follow: 5′-CACTTATAGAGAAGTTGAATT-3′ (Sigma).

### RT-PCR

Total RNA was isolated from HeLa cells using a TRIzol reagent (Ambion) and synthesis first-strand cDNA was performed using the PrimeScript RT reagent Kit (Takara Bio). Quantitative real-time RT-PCR was performed in an Mx3000 P QPCR system (Agilent Technologies) using SYBR Premix Ex Taq^TM^ II (Ti RNaseH Plus) (Takara Bio). Primers used were as follows: human *RPL3* sense: Hs*RPL*3.Fw.5′-CCGCACTGAGATCAACAAGA-3′ antisense: Hs*RPL3*.Rv. 5′-CAGCCTTTCAGCATGACAAA-3′, human *DHRS7B* sense: *DHRS7B*355Fw. 5′-TGACCTTCGACC TCACAGAC-3′ antisense: DHRS7B484 Rv. 5′-CCCTCTTGT CCACATCCAACT-3′.

### Plasmid Construction

The following plasmids used were as described: pcDNAZeo/HA_2_-*PEX19*, pcDNAZeo/HA_2_-*PEX19*ΔN23 (Matsuzono et al., 2006), and pcDNAZeo/FLAG-*PEX26* (Yagita et al., 2013). pcDNAZeo/FLAG-NLS-*PEX19* containing three contiguous copies of the viral SV-40 T antigen nuclear localization signal (Adam and Gerace, 1991) was generated by inverse PCR using a plasmid encoding FLAG-*PEX19* fused with single NLS between FLAG and *PEX19* coding sequences (Koyama, Yagita, and Fujiki unpublished). The cDNAs encoding Sec61βC (amino acid sequence at positions 71–96) was amplified by PCR and cloned into pcDNAZeo/EGFP (Yagita et al., 2013) via the *Bam*HI–*Not*I site. The cDNAs encoding full-length ADHAPR was amplified by PCR using the RT-product as a template with a set of primers, HsDHRS7B Fw: 5′-GGATCCGCCACCATGGTCTCTCCGGCTACC-3′ and HsDHRS7B Rv: 5′-CCTCGAGCTAGGAGTTCTTGGATTTC-3′, and cloned into pcDNA3.1/Zeo (+) vector (Invitrogen) between *Bam*HI–*Xho*I site. The resultant plasmid was used for a template for tagging FALG tag by PCR using a sets of primers, FLAG7B inv. Fw: 5′-GACGATAAGGGCGGTGTCTCTCCGGCTACCAGGA-3′ and FLAG7Binv.Rv: 5′- GTCGTCCTTGTAATCCATGGTGGCGT CTCCCTATA-3′, to generate pcDNAZeo/FLAG-*ADHAPR*. The deletion mutant of ΔN16 and the glycosylation site mutant (P4N) were generated from pcDNAZeo/FLAG-*ADHAPR* using overlap extension PCR (Ho et al., 1989).

### Immunoblotting

Immunoblotting was performed as described (Otera et al., 2000). In brief, protein samples were separated by SDS-PAGE and electrotransferred to a polyvinylidene fluoride membrane (Bio-rad Laboratories). After blocking in PBS containing 5% non-fat dry milk and 0.1% Tween 20, blots were subjected to immunoblotting with the following antibodies: rabbit polyclonal antibodies to ADHAPR (ABGENT.COM), protein disulfide isomerase (PDI) (Stressgen), acyl-CoA oxidase (AOx; raised against full-length rat AOx) (Tsukamoto et al., 1990), Pex14p (Shimizu et al., 1999), Pex3p (Ghaedi et al., 2000), and influenza hamagglutinin epitope (YPYDVPDYA) (Otera et al., 2000). The following primary antibodies were purchased from the indicated vendors; mouse monoclonal antobodies to FLAG (M2; Sigma-Aldrich), α-tubulin (Thermo Fisher Scientific), HA (16B12; Covance), BiP (Transduction laboratory), and cytochrome P450 reductase, GFP, and SRP54 (Santa Cruz Biotechnology, Inc.) and goat anti-lactate dehydrogenase antibody (LDH) (Rockland). After probing with appropriate HRP-conjugated secondary antibodies, immunoblots were developed with ECL Western blotting detection reagents (GE Healthcare), and scanned with an LAS-4000 Mini luminescent image analyzer (Fujifilm).

### Immunofluorescence Microscopy

Cells on glass coverslips were fixed with 4% paraformaldehyde in PBS for 15 min at RT, permeabilized with 1% Triton X-100 in PBS for 5 min at RT, and blocked with PBS-BSA (PBS containing 1% BSA) for 60 min at RT. Subsequently, cells were incubated for 120 min at RT with primary antibodies diluted in PBS-BSA. Primary antibodies used were as followings; rabbit polyclonal antibody to calnexin (Stressgen), mouse monoclonal antibodies to ADHAPR (ABNOVA) and KDEL (Stressgen), and guinea pig anti-Pex14p antibody (Mukai et al., 2002). Antigen-antibody complexs were visualized using Alexa Fluor conjugated sencondary antibodies and observed as described (Honsho et al., 2017). Analysis of colocalization of Pex14p with FLAG-ADHAPR and FLAG-ΔN16ADHAPR in FAA.K1B cells was assessed by staining with guinea pig anti-Pex14p antibody and mouse monoclonal antibody to FLAG, followed by determining the colocalization using ZEN 2012 imaging software (Carl Zeiss).

### *In vitro* Import Assay Using Digitonin-Permeabilized HeLa Cells

HeLa cells were permeabilized as described (Matsuzaki and Fujiki, 2008) using 50 μg/ml digitonin in buffer S (0.25 M sucrose, 25 mM Hepes-KOH, pH 7.4, 2.5 mM magnesium acetate, 2.5 mM KCl, 2 mM EGTA, 0.01% taxol, and protease inhibitor cocktail [5 μg/ml aprotinin and 10 μg/ml each of antipain, chymostatin, E-64, leupeptin, and pepstatin]).

Semi-permeabilized HeLa cells were incubated for 60 min at 26°C with *in vitro*–synthesized proteins in buffer S as described (Yagita et al., 2013). After extensive washing, cells were subjected to the immunofluorescence microscopy procedure described above.

### Subcellular Fractionation

Cells were collected in buffer H (0.25 M sucrose, 20 mM Hepes-KOH, pH 7.4, 1 mM EDTA, and a protease inhibitor cocktail) and homogenized on ice by passing through a 27-gauge needle (with 1 ml syringe). Homogenates were centrifuged at 800 × *g* for 5 min to yield a postnuclear supernatant fraction (PNS). The PNS was then separated into cytosolic and organelle fractions by ultracentrifugation at 100,000 × *g* for 30 min (Honsho et al., 2013).

For alkaline extraction (Fujiki et al., 1982), the PNS fractions were treated with 0.1 M Na_2_CO_3_ on ice for 30 min, and soluble and membrane fractions were separated by ultracentrifugation at 100,000 × *g* for 30 min.

For proteinase K-sensitivity assay, HeLa cells were collected in buffer H (-protease inhibitor) and prepared PNS. The PNS was treated with 50 μg/ml proteinase K (Sigma-Aldrich) for 30 min on ice, followed by adding PMSF (Nacalai Tesque) to terminate the reaction.

### Mutation Analysis

Total RNA was prepared from CHO-K1 and FAA.K1B cells using a TRIzol reagent (Ambion) and synthesis of first-strand cDNA was performed using the PrimeScript RT reagent Kit (Takara Bio). The entire open reading frame of *DHRS7B* was amplified using a set of primers, a sense *Kpn*I choADHAPRFw: 5′-AAGGTACCTTTACGTCAATTCCGA-3′ and an anti-sense choADHAPR-*Apa*I Rv2: 5′-TTGGGCCCTAGCAGCTCTGAGC-3′. The PCR fragments were digested with *Kpn*I and *Apa*I and were ligated between the *Kpn*I and *Apa*I sites of pcDNA3.1/Zeo vector. The nucleotide sequence of *DHRS7B* from CHO-K1 and FAA.K1B was determined from six each independent clones.

### Lipid Analysis

Cells were cultured for 2 h in the presence of 0.1 μCi^/^ml of ^14^C-Etn. Equal aliquots (100 μg protein) of cell lysates were treated with 5% of trichloroacetic acid for 10 min at room temperature and precipitated by 20,000 × *g* for 1 min, followed by lipid extraction by the Bligh and Dyer method (Bligh and Dyer, 1959). Lipids were analyzed on TLC plates (silica gel 60, Merck) with chloroform/methanol/acetic acid solution (v/v/v: 65/25/10) (Honsho et al., 2008). ^14^C-labeled lipids were detected by autoradiography using a FLA-5000 imaging analyzer and quantified using an image analyser software (Multi Gauge, Fuji Film).

### Statistical Analysis and Data Presentation

Statistical analysis was performed using one-tailed Student’s *t*-tests unless otherwise described in figure legends. A *P* < 0.05 was considered statistically significant. Quantitative data were shown as mean ± SD.

### Other Methods

Immunoprecipitation from cytosolic fractions prepared from *pex19* CHO ZP119 cells co-expressing HA_2_-Pex19p and either FLAG-ADHARP or FLAG-Pex26p was performed as described (Yagita et al., 2013). Endoglycosidase H digestion was carried out according to the manufacturer’s instructions (New England Biolabs).

## Data Availability Statement

The raw data supporting the conclusions of this article will be made available by the authors, without undue reservation, to any qualified researcher.

## Author Contributions

MH conceived and designed the study. MH and MT performed the experiments and interpreted the data. RZ provided resources and edited the manuscript. MH and YF wrote and edited the manuscript. All authors contributed to the article and approved the submitted version.

## Conflict of Interest

The authors declare that the research was conducted in the absence of any commercial or financial relationships that could be construed as a potential conflict of interest. The reviewer KW declared a past collaboration with one of the authors RZ to the handling editor.

## Abbreviations

ADAPS: alkyl-dihydroxyacetonephosphate synthase
ADHAPR: acyl/alkyl DHAP reductase
CHO: Chinese hamster ovary
DHAPAT: dihydroxyacetonephosphate acyltransferase
ER: endoplasmic reticulum
Etn: ethanolamine
NLS: nuclear localization signal
Pex: peroxin
PexRAP: peroxisomal reductase-activating PPAR γ
PlsEtn: ethanolamine plasmalogen
PMP: peroxisomal membrane protein
PNS: postnuclear supernatant
PtdEtn: phosphatidylethanolamine
TMD: transmembrane domain
RRL: rabbit reticulocyte lysate
SRP: signal recognition particle.

## References

[B1] Adam S. A. Gerace L. (1991). Cytosolic proteins that specifically bind nuclear location signals are receptors for nuclear import. *Cell* 66 837–847. 10.1016/0092-8674(91)90431-w 1653647

[B2] Aviram N. Ast T. Costa E. A. Arakel E. C. Chuartzman S. G. Jan C. H. (2016). The SND proteins constitute an alternative targeting route to the endoplasmic reticulum. *Nature* 540 134–138. 10.1038/nature20169 27905431 PMC5513701

[B3] Bligh W.J. Dyer L. (1959) A rapid method of total lipid extraction and purification. *Can. J. Biochem. Physiol.* 37 911–917. 13671378 10.1139/o59-099

[B4] Borgese N. Fasana E. (2011). Targeting pathways of C-tail-anchored proteins. *Biochim. Biophys. Acta* 1808 937–946. 10.1016/j.bbamem.2010.07.010 20646998

[B5] Boström K. Wettesten M. Borén J. Bondjers G. Wiklund O. Olofsson S. O. (1986). Pulse-chase studies of the synthesis and intracellular transport of apolipoprotein B-100 in Hep G2 cells. *J. Biol. Chem.* 261 13800–13806. 3020051

[B6] Braverman N. E. Moser A. B. (2012). Functions of plasmalogen lipids in health and disease. *Biochim. Biophys. Acta.* 1822 1442–1452. 10.1016/j.bbadis.2012.05.008 22627108

[B7] Filling C. Berndt K. D. Benach J. Knapp S. Prozorovski T. Nordling E. (2002). Critical residues for structure and catalysis in short-chain dehydrogenases/reductases. *J. Biol. Chem.* 277 25677–25684. 10.1074/jbc.m202160200 11976334

[B8] Fujiki Y. Hubbard A. L. Fowler S. Lazarow P. B. (1982). Isolation of intracellular membranes by means of sodium carbonate treatment: application to endoplasmic reticulum. *J. Cell Biol.* 93 97–102. 10.1083/jcb.93.1.97 7068762 PMC2112113

[B9] Gallego-García A. Monera-Girona A. J. Pajares-Martínez E. Bastida-Martínez E. Pérez-Castaño R. Iniesta A. A. (2019). A bacterial light response reveals an orphan desaturase for human plasmalogen synthesis. *Science* 366 128–132. 10.1126/science.aay1436 31604315

[B10] Ghaedi K. Kawai A. Okumoto K. Tamura S. Shimozawa N. Suzuki Y. (1999). Isolation and characterization of novel peroxisome biogenesis-defective Chinese hamster ovary cell mutants using green fluorescent protein. *Exp. Cell Res.* 248 489–497. 10.1006/excr.1999.4413 10222140

[B11] Ghaedi K. Tamura S. Okumoto K. Matsuzono Y. Fujiki Y. (2000). The peroxin Pex3p initiates membrane assembly in peroxisome biogenesis. *Mol. Biol. Cell* 11 2085–2102. 10.1091/mbc.11.6.2085 10848631 PMC14905

[B12] Ghosh M. K. Hajra A. K. (1986). Subcellular distribution and properties of acyl/alkyl dihydroxyacetone phosphate reductase in rodent livers. *Arch. Biochem. Biophys.* 245 523–530. 10.1016/0003-9861(86)90245-6 3954368

[B13] Hajra A. K. Larkins L. K. Das A. K. Hemati N. Erickson R. L. MacDougald O. A. (2000). Induction of the peroxisomal glycerolipid-synthesizing enzymes during differentiation of 3T3-L1 adipocytes. Role in triacylglycerol synthesis. *J. Biol. Chem.* 275 9441–9446. 10.1074/jbc.275.13.9441 10734090

[B14] Haßdenteufel S. Sicking M. Schorr S. Aviram N. Fecher-Trost C. Schuldiner M. (2017). hSnd2 protein represents an alternative targeting factor to the endoplasmic reticulum in human cells. *FEBS Lett.* 591 3211–3224. 10.1002/1873-3468.12831 28862756

[B15] Hegde R. S. Keenan R. J. (2011). Tail-anchored membrane protein insertion into the endoplasmic reticulum. *Nat. Rev. Mol. Cell Biol.* 12 787–798. 10.1038/nrm3226 22086371 PMC3760496

[B16] Ho S. N. Hunt H. D. Horton R. M. Pullen J. K. Pease L. R. (1989). Site-directed mutagenesis by overlap extension using the polymerase chain reaction. *Gene* 77 51–59. 10.1016/0378-1119(89)90358-2 2744487

[B17] Honsho M. Abe Y. Fujiki Y. (2017). Plasmalogen biosynthesis is spatiotemporally regulated by sensing plasmalogens in the inner leaflet of plasma membranes. *Sci. Rep.* 7:43936. 10.1038/srep43936 PMC5341075 28272479

[B18] Honsho M. Asaoku S. Fukumoto K. Fujiki Y. (2013). Topogenesis and homeostasis of fatty acyl-CoA reductase 1. *J. Biol. Chem.* 288 34588–34598. 10.1074/jbc.m113.498345 24108123 PMC3843072

[B19] Honsho M. Yagita Y. Kinoshita N. Fujiki Y. (2008). Isolation and characterization of mutant animal cell line defective in alkyl-dihydroxyacetonephosphate synthase: localization and transport of plasmalogens to post-Golgi compartments. *Biochim. Biophys. Acta* 1783 1857–1865. 10.1016/j.bbamcr.2008.05.018 18571506

[B20] Huang T. Y. Zheng D. Hickner R. C. Brault J. J. Cortright R. N. (2019). Peroxisomal gene and protein expression increase in response to a high-lipid challenge in human skeletal muscle. *Metabolism* 98 53–61. 10.1016/j.metabol.2019.06.009 31226353 PMC7031862

[B21] Ingolia N. T. Lareau L. F. Weissman J. S. (2011). Ribosome profiling of mouse embryonic stem cells reveals the complexity and dynamics of mammalian proteomes. *Cell* 147 789–802. 10.1016/j.cell.2011.10.002 22056041 PMC3225288

[B22] James P. F. Lake A. C. Hajra A. K. Larkins L. K. Robinson M. Buchanan F. G. (1997). An animal cell mutant with a deficiency in acyl/alkyl-dihydroxyacetone-phosphate reductase activity. Effects on the biosynthesis of ether-linked and diacyl glycerolipids. *J. Biol. Chem.* 272 23540–23546. 10.1074/jbc.272.38.23540 9295290

[B23] James P. F. Zoeller R. A. (1997). Isolation of animal cell mutants defective in long-chain fatty aldehyde dehydrogenase. Sensitivity to fatty aldehydes and schiff’s base modification of phospholipids: implications for sjögren-larsson syndrome. *J. Biol. Chem.* 272 23532–23539. 10.1074/jbc.272.38.23532 9295289

[B24] Jones J. M. Morrell J. C. Gould S. J. (2004). PEX19 is a predominantly cytosolic chaperone and import receptor for class 1 peroxisomal membrane proteins. *J. Cell Biol.* 164 57–67. 10.1083/jcb.200304111 14709540 PMC2171958

[B25] Jörnvall H. Persson B. Krook M. Atrian S. Gonzàlez-Duarte R. Jeffery J. (1995). Short-chain dehydrogenases/reductases (SDR). *Biochemistry* 34 6003–6013. 10.1021/bi00018a001 7742302

[B26] Kanda S. Yanagitani K. Yokota Y. Esaki Y. Kohno K. (2016). Autonomous translational pausing is required for XBP1u mRNA recruitment to the ER via the SRP pathway. *Proc. Natl. Acad. Sci. U.S.A.* 113 E5886–E5895. 27651490 10.1073/pnas.1604435113 PMC5056097

[B27] Karamyshev A. L. Patrick A. E. Karamysheva Z. N. Griesemer D. S. Hudson H. Tjon-Kon-Sang S. (2014). Inefficient SRP interaction with a nascent chain triggers a mRNA quality control pathway. *Cell* 156 146–157. 10.1016/j.cell.2013.12.017 24439374 PMC3931426

[B28] Kashiwayama Y. Asahina K. Morita M. Imanaka T. (2007). Hydrophobic regions adjacent to transmembrane domains 1 and 5 are important for the targeting of the 70-kDa peroxisomal membrane protein. *J. Biol. Chem.* 282 33831–33844. 10.1074/jbc.m703369200 17761678

[B29] Kinoshita N. Ghaedi K. Shimozawa N. Wanders R. J. A. Matsuzono Y. Imanaka T. (1998). Newly identified Chinese hamster ovary cell mutants are defective in biogenesis of peroxisomal membrane vesicles (peroxisomal ghosts), representing a novel complementation group in mammals. *J. Biol. Chem.* 273 24122–24130. 10.1074/jbc.273.37.24122 9727033

[B30] LaBelle E. F. J. Hajra A. K. (1974). Purification and kinetic properties of acyl and alkyl dihydroxyacetone phosphate oxidoreductase. *J. Biol. Chem.* 249 6936–6944. 4153765

[B31] Lee J. H. Chandrasekar S. Chung S. Hwang Fu Y. H. Liu D. Weiss S. (2018). Sequential activation of human signal recognition particle by the ribosome and signal sequence drives efficient protein targeting. *Proc. Natl. Acad. Sci. U.S.A.* 115 E5487–E5496. 29848629 10.1073/pnas.1802252115 PMC6004459

[B32] Liu Y. Yagita Y. Fujiki Y. (2016). Assembly of peroxisomal membrane proteins via the direct Pex19p-Pex3p pathway. *Traffic* 17 433–455. 10.1111/tra.12376 26777132

[B33] Lodhi I. J. Dean J. M. He A. Park H. Tan M. Feng C. (2017). PexRAP Inhibits PRDM16-mediated thermogenic gene expression. *Cell Rep.* 20 2766–2774. 10.1016/j.celrep.2017.08.077 28930673 PMC5679740

[B34] Lodhi I. J. Yin L. Jensen-Urstad A. P. L. Funai K. Coleman T. Baird J. H. (2012). Inhibiting adipose tissue lipogenesis reprograms thermogenesis and PPARγ activation to decrease diet-induced obesity. *Cell Metab.* 16 189–201. 10.1016/j.cmet.2012.06.013 22863804 PMC3467338

[B35] Matsumoto N. Tamura S. Fujiki Y. (2003). The pathogenic peroxin Pex26p recruits the Pex1p-Pex6p AAA ATPase complexes to peroxisomes. *Nat. Cell Biol.* 5 454–460. 10.1038/ncb982 12717447

[B36] Matsuzaki T. Fujiki Y. (2008). The peroxisomal membrane protein import receptor Pex3p is directly transported to peroxisomes by a novel Pex19p- and Pex16p-dependent pathway. *J. Cell Biol.* 183 1275–1286. 10.1083/jcb.200806062 19114594 PMC2606968

[B37] Matsuzono Y. Kinoshita N. Tamura S. Shimozawa N. Hamasaki M. Ghaedi K. (1999). Human PEX19: cDNA cloning by functional complementation, mutation analysis in a patient with Zellweger syndrome, and potential role in peroxisomal membrane assembly. *Proc. Natl. Acad. Sci. U.S.A.* 96 2116–2121. 10.1073/pnas.96.5.2116 10051604 PMC26746

[B38] Matsuzono Y. Matsuzaki T. Fujiki Y. (2006). Functional domain mapping of peroxin Pex19p: interaction with Pex3p is essential for function and translocation. *J. Cell Sci.* 119 3539–3550. 10.1242/jcs.03100 16895967

[B39] Mukai S. Ghaedi K. Fujiki Y. (2002). Intracellular localization, function, and dysfunction of the peroxisome-targeting signal type 2 receptor, Pex7p, in mammalian cells. *J. Biol. Chem.* 277 9548–9561. 10.1074/jbc.m108635200 11756410

[B40] Nagan N. Zoeller R. A. (2001). Plasmalogens: biosynthesis and functions. *Prog. Lipid Res.* 40 199–229. 10.1016/s0163-7827(01)00003-0 11275267

[B41] Neve E. P. Ingelman-Sundberg M. (2008). Intracellular transport and localization of microsomal cytochrome P450. *Anal. Bioanal. Chem.* 392 1075–1084. 10.1007/s00216-008-2200-z 18537026

[B42] Otera H. Harano T. Honsho M. Ghaedi K. Mukai S. Tanaka A. (2000). The mammalian peroxin Pex5pL, the longer isoform of the mobile peroxisome targeting signal (PTS) type 1 transporter, translocates Pex7p-PTS2 protein complex into peroxisomes via its initial docking site, Pex14p. *J. Biol. Chem.* 275 21703–21714. 10.1074/jbc.m000720200 10767286

[B43] Sakaue H. Iwashita S. Yamashita Y. Kida Y. Sakaguchi M. (2016). The N-terminal motif of PMP70 suppresses cotranslational targeting to the endoplasmic reticulum. *J. Biochem.* 159 539–551. 10.1093/jb/mvv132 26711236

[B44] Schrul B. Kopito R. R. (2016). Peroxin-dependent targeting of a lipid-droplet-destined membrane protein to ER subdomains. *Nat. Cell Biol.* 18 740–751. 10.1038/ncb3373 27295553 PMC4925261

[B45] Shimizu N. Itoh R. Hirono Y. Otera H. Ghaedi K. Tateishi K. (1999). The peroxin Pex14p: cDNA cloning by functional complementation on a Chinese hamster ovary cell mutant, characterization, and functional analysis. *J. Biol. Chem.* 274 12593–12604. 10.1074/jbc.274.18.12593 10212238

[B46] Thai T.-P. Heid H. Rackwitz H.-R. Hunziker A. Gorgas K. Just W. W. (1997). Ether lipid biosynthesis: isolation and molecular characterization of human dihydroxyacetonephosphate acyltransferase. *FEBS Lett.* 420 205–211. 10.1016/s0014-5793(97)01495-6 9459311

[B47] Tsukamoto T. Yokota S. Fujiki Y. (1990). Isolation and characterization of Chinese hamster ovary cell mutants defective in assembly of peroxisomes. *J. Cell Biol.* 110 651–660. 10.1083/jcb.110.3.651 1689731 PMC2116037

[B48] Walter P. Johnson A. E. (1994). Signal sequence recognition and protein targeting to the endoplasmic reticulum membrane. *Annu. Rev. Cell Biol.* 10 87–119. 10.1146/annurev.cb.10.110194.000511 7888184

[B49] Werner E. R. Keller M. A. Sailer S. Lackner K. Koch J. Hermann M. (2020). The TMEM189 gene encodes plasmanylethanolamine desaturase which introduces the characteristic vinyl ether double bond into plasmalogens. *Proc. Natl. Acad. Sci. U.S.A.* 117 7792–7798. 10.1073/pnas.1917461117 32209662 PMC7149458

[B50] Yagita Y. Hiromasa T. Fujiki Y. (2013). Tail-anchored PEX26 targets peroxisomes via a PEX19-dependent and TRC40-independent class I pathway. *J. Cell Biol.* 200 651–666. 10.1083/jcb.201211077 23460677 PMC3587837

