# Supplementary material for: Distinct Functions of Acyl/Alkyl Dihydroxyacetonephosphate Reductase in Peroxisomes and Endoplasmic Reticulum
Source: Front Cell Dev Biol. 2020 Sep 11;8:855. doi: 10.3389/fcell.2020.00855 (PMC7517302; doi:10.3389/fcell.2020.00855)
Supplement: Supplementary file 1 [file Image_1.pdf]

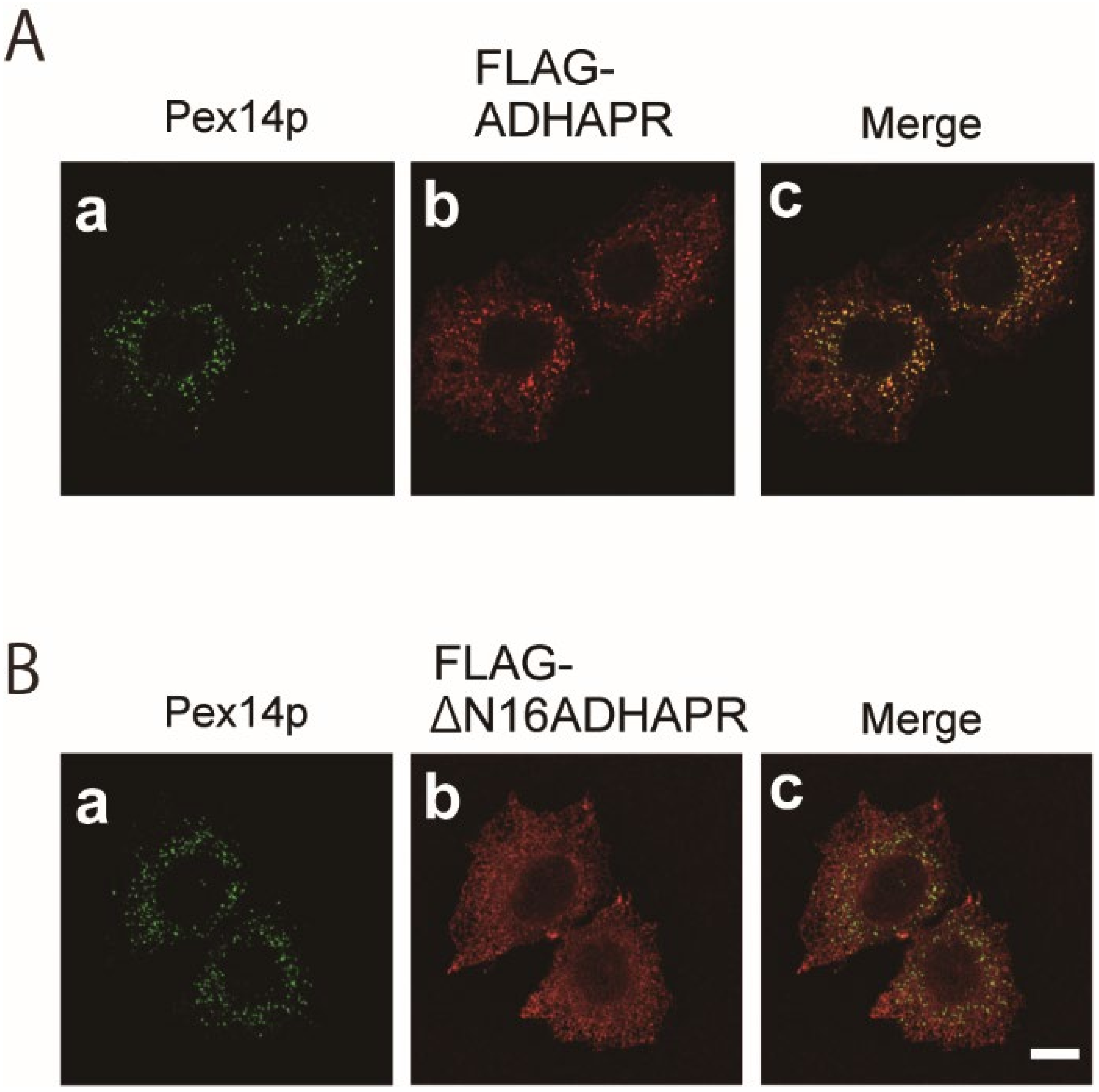

Figure S1. Immunofluorescent images of FAA.K1B cells stably expressing FLAG-ADHAPR and FLAG-ΔN16ADHAPR.
A) Intracellular localization of FLAG-ADHAPR was assessed in the FAA.K1B cell line stably expressing FLAG-ADHAPR by the immunofluorescence staining. FLAG-ADHAPR was verified with monoclonal anti-FLAG antibody (b) and peroxisomes were visualized by immunostaining with anti-Pex14p antibody (a), respectively. Merged views are shown in (c). B) Intracellular localization of FLAG-ΔN16ADHAPR was likewise assessed. Bar, 10 µm.
